# *Bifidobacterium lactis* Ameliorates the Risk of Food Allergy in Chinese Children by Affecting Relative Percentage of Treg and Th17 Cells

**DOI:** 10.1155/2018/4561038

**Published:** 2018-12-11

**Authors:** Qingbin Liu, Wei Jing, Wei Wang

**Affiliations:** Department of Pediatric, Affiliated Hospital of Changchun University of Traditional Chinese Medicine, Changchun 130021, China

## Abstract

We aimed to explore the therapeutic effect of *Bifidobacterium lactis* on food allergy by investigating the percentage of Treg and Th17 cells in Chinese children and related molecular mechanisms. A total of 256 children with food allergy were evenly assigned into two groups: BG, the children received 10 ml *B. lactis* (1 × 10^6^/ml) daily, and CG, the children received the solution without *B. lactis* daily for three months. Allergic symptoms, serum IgE, and food antigen-specific IgE were measured. A mouse allergy model was established by using shrimp tropomyosin and treated with *B. lactis*. Relative mRNA levels of Treg- and Th17-associated cytokines were measured by using quantitative PCR. The percentage of Treg and Th17 cells in spleen were measured by using flow cytometry. After 3-month therapy, the allergic symptoms of the BG were remarkably reduced when compared with the CG (*P* < 0.05). Serum levels of IgE and food antigen-specific IgE were decreased too (*P* < 0.05). Similar results were also found in a mouse allergy model. After *B. lactis* treatment, the relative mRNA level of FoxP3 was significantly enhanced in the *B. lactis* therapy group when compared to positive controls. In addition, relative mRNA levels of FoxP3 and TGF-*β* associated with Treg cells were increased, whereas relative mRNA levels of IL-17A and IL-23 associated with Th17 were reduced. *B. lactis* treatment significantly increased the ratio of Treg and Th17 cells in a mouse allergy model (*P* < 0.05). *B. lactis* effectively alleviates allergic symptoms by increasing the ratio of Treg and Th17 cells.

## 1. Introduction

The prevalence of food allergy (FA) has significantly increased in the pediatric population. However, the effective treatment is still lacking [1]. The etiology of food allergy is complex with individual differences in different patients [2]. The pathogenesis of food allergy involves many aspects, including immunity [3], genetics [4], *Helicobacter pylori* infection [5], and environment [6].

Food allergens are the main reason for causing allergic disorders. Among shellfish family, crustacean food can cause a large amount of allergic responses. For instance, shrimp has high-level tropomyosin, which is the factor for causing food allergies [7]. Shrimp [8] and crab [9] allergens are among the major types of food allergens reported by the Food and Agriculture Organization (FAO). Both crustaceans have tropomyosin [10], arginine kinase [11, 12], myosin [13, 14], and sarcoplasmic calcium-binding protein allergens [15, 16]. The incidence of crustacean allergy is very common with 38% in food allergy [17]. In crustaceans, shrimp is favored by most consumers worldwide because it is delicious and rich in nutrients; however, shrimp causes the most allergic reaction with more than 50% of crustacean allergy. The prevalence of crustacean allergy is varied in different geographical locations [18]. Many studies have found that the allergic reaction is mainly Th2-induced inflammatory response [19]. Recently, it has been found that the imbalance between regulatory T cells (Treg) and Th17 in T-lymphocyte subsets also leads to immune disorders [20].

Th17 and Treg cells are the two main subsets of CD4^+^ T cells and participate in allergy responses [21]. FoxP3 is an intracellular transcription factor of Treg cells [22]. TGF-*β* plays a critical role in Treg cell development and can affect the number and function of Treg cells [23]. IL-17A is an important factor that accompanies pathogen infection and mainly produced by Th17 cells [24]. IL-23 is another important cytokine associated with Th17 cells [25]. Both IL-17 and IL-23 can affect the balance of Th17/Treg cells [26]. Thus, these cytokines were measured in mouse allergy models.

Treg cells can avoid excessive immune response by activating T cells expressing IL-2 receptor alpha-chains (CD25) and secreting cytokines such as IL-10 and TGF-*β* [27]. Th17 cells are a new type of CD4^+^ T cells [28] and mainly exert inflammatory action by secreting orphan nuclear receptor yt (RORyt) and other effector cytokines such as IL-17A and IL-6, which can induce allergic asthma, systemic lupus erythematosus, rheumatoid arthritis, and other allergic diseases [29, 30]. A strong Th17-type immune response is induced, and a large number of inflammatory cytokines (IL-17, IL-6, IL-23, etc.) will be released [31, 32] when a food allergic reaction occurs.

Probiotics, as a kind of beneficial active microorganisms, have been fully affirmed in immune function by generating short-chain fatty acids [33], polysaccharides [34, 35], and cell wall components (peptidoglycan and lipoteichoic acid) [36]. Previous studies indicated that *Bifidobacterium* regulated the components of gut microbiota. The changes in *Dorea* and *Ralstonia* bacteria were closely associated with the Th2/Treg ratio and contributed to the reduction of tropomyosin-induced allergic responses. *Bifidobacterium* can alleviate food allergy and regulate gut immune homeostasis [37]. *Bifidobacterium lactis* is one of the main probiotics in yogurt, and it is safe and well tolerated [38]. *B. lactis* has been approved to be effective in the prevention of ovalbumin (OVA)-induced allergy in a mouse model [39]. However, its mechanism remains unclear. In the present study, we investigated the therapeutic effect of *B. lactis* on the allergic reaction in infants, the percentage of Treg and Th17 cells, and related cytokines to provide theoretical basis in the prevention of food allergy.

## 2. Materials and Methods

### 2.1. Participants

Before the present experiment, all procedures were approved by the Human Research Committee of Changchun University of Traditional Chinese Medicine (Changchun, China). The children's parents and caretakers were ready to help all children strictly adhere to all protocols and signed informed consent form. Intent-to-treat (ITT) population and the per-protocol (PP) population were the same.

### 2.2. Allergy Skin Test

Hypersensitivity response of each patient was assessed by using conventional skin prick tests against 16 common aeroallergens according to an earlier report [40]. Skin prick tests were performed according to the methods introduced by Gislason and Gislason [41]. The test would be regarded as clinically significant if allergens reactions were more than 10 among 16 common aeroallergens.

### 2.3. Measurement of Serum IgE

Serum IgE is an important indicator to determine food and aeroallergen sensitization in the children with food allergy [42]. Thus, serum IgE was measured in the children with food allergy. 3 ml venous blood was obtained from each patient, and serum was isolated via centrifugation. Serum IgE was measured by using ELISA kits from Thermo Fisher Scientific (Cat. no.: 88-50610-22, Carlsbad, CA, USA).

### 2.4. Measurement of Allergic Reactions on Food-Specific IgE of Food

Antigen-specific IgE that specifically binds to food allergens was measured by using the allergen diagnostic kit (MEDIWISS Analytic GmbH, Moers, Germany). This kit could be used to detect antigen-specific IgE of 9 kinds of food allergens in human serum, including egg, milk, shrimp, beef, shellfish, crab, mango, cashew nuts, and pineapple. Food-specific allergen was adsorbed on the surface of nitrocellulose membrane and placed on the reaction tank. The allergen-specific IgE antibody in the sample reacted with the allergen and bound to the nitrocellulose membrane. After excess antibody was eluted, biotin-labeled anti-human IgE antibody was added and incubated at room temperature. Alkaline phosphatase-labeled streptavidin was added and incubated at room temperature. After the addition of the BCIP/NBT substrate, a specific enzyme color reaction occurred and a precipitate appeared on the strip. The color intensity was proportional to specific antibody content of the serum sample and serum IgE. The color intensity was measured by using a densitometer (BioRad, Hercules, CA, USA). If the OD_490nm_ values were more than two by the immunodot method, the food allergen-specific IgE reaction was regarded as positive.

### 2.5. Inclusion Criteria

The following patients were included: the patients who were clinically diagnosed with food allergy and a history of food allergy more than 1 year; the patients who were aged from 4 to 12 years; allergen-specific IgE responses that were more than 10 in food allergy test (20 allergen-specific IgEs could be tested each time). Total serum IgE levels were more than 4.5 *μ*g/ml. The patients had normal food allergies, such as cutaneous manifestation (rash and eczema) and gastrointestinal symptoms (abdominal cramps, abdomen pain, and vomiting). The severity of atopic rash was measured by using the National Cancer Institute Common Terminology Criteria for Adverse Events version 3.0 (NCI CTCAE v3.0) (grades 1–5) [43]. Atopic eczema was measured by a dermatologist by using severity scoring of atopic dermatitis (SCORAD) (scores 0–103) [44]. Abdominal cramps and pain were measured by electrolyte disturbance (scores 0–45) [45]. Incidence of nausea and vomiting was measured using a 4-point scale (0 = asymptomatic, 1 = mild (subjective nausea was present and recovery was achieved without drug treatment), 2 = moderate (subjective nausea was present and recovery was achieved using antiemetics), and 3 = severe (subjective symptoms such as nausea and vomiting were present, and gastric contents were released)) by selecting the highest one at the time when the measurement was performed [46]. The patients had more than 6 events of food allergy (rash and eczema, abdominal cramps, abdomen pain, vomiting, and so on) within one month, and the symptoms duration was more than half an hour at each time.

### 2.6. Exclusion Criteria

The following patients were excluded: the patients had pharmacological and medical therapy within three months, including vitamin, minerals, glucocorticoid drugs, and antibiotics; the patients were allergic to yogurt or dairy products; the patients had cardiac failure, renal failure, or other serious organ disorders; and the patients had irritable bowel syndrome (IBS) and inflammatory bowel diseases (IBD).

### 2.7. Study Intervention

*B. lactis* yogurt culture was purchased from Inner Mongolia Yili Industrial Group Company (Yili, China). The strain was isolated, identified by 16S rRNA, and inoculated in the MRS medium (Hangzhou Shuangtian Biological Co., Ltd.) for 24 h at 37°C in anaerobic environment. The bacterial suspension was centrifuged at 2000 ×*g* at 4°C for 10 min, pelleted with PBS (phosphate buffer saline) at the concentration of 10^9^ CFU/ml, and stored at 4°C. A total of 256 children with food allergy were evenly assigned into two groups in a double-blind clinical study: *B. lactis* group (BG, those who received 10 ml *B. lactis* (1 × 10^6^/ml) daily, *n* = 128) and control group (CG, those who received the solution without *B. lactis*, *n* = 128).

The study duration was three months. Allergic children meeting exclusion and inclusion criteria were recruited in the present experiment. The detailed history of patients was obtained from his or her guider and examined by two experts. All subjects were inquired to write down any unwanted adverse effects in the 3-month follow-up. All patients were visited before this study as the first visit, after one month as the second visit, and after 3-month follow-up as a final visit. Allergy symptoms were recorded at each time.

### 2.8. Establishment of Allergy Model

Before the present experiment, all procedures were approved by the animal research committee of Changchun University of Traditional Chinese Medicine (approval no. 20161206XYZ). Tropomyosin of Chinese shrimp (*Penaeus orientalis*) was purified according to a previous report [47]. BALB/c mice (4 weeks old, 18–22 g) were purchased from the animal center of the Changchun University of Traditional Chinese Medicine and raised in our animal facility. The mice were maintained at 20–22°C under a 12-h light and 12-h dark condition with free access to food and water. A total of 24 BALB/c mice were randomly divided into 3 groups (*n* = 8 in each group): negative control group (NG), positive control group (PG), and experiment group (EG). After one-week acclimatization, the mice from PG and EG were intracutaneously injected with 100-*μ*g tropomyosin and 10-*μ*g cholera toxin (Sigma) as an adjuvant in 0.1 ml of PBS buffer twice weekly for a month. The mice from NG were intracutaneously injected with 10-*μ*g cholera toxin in PBS buffer twice weekly for a month. Subsequently, the mice from EG were orally administrated with 0.5-ml (1 × 10^6^/ml) *B. lactis* in 0.9% saline solution for 28 d. The mice from NG and PG received equal volume of saline solution. After 28 d, the mice were sacrificed by cervical dislocation. The spleen was isolated and exteriorized.

### 2.9. Evaluation of Systemic Anaphylaxis in Mouse Allergy Models

The symptoms of systemic anaphylaxis were measured before and after tropomyosin challenge and scored based on a scoring system [48]: 0, no symptoms; 1, scratch and rub nose and head; 2, swelling eyes and mouth, diarrhea, slow or no activity with an increased respiratory rate after prodding; 3, wheezing and cyanosis around the mouth and the tail; 4, no activity; and 5, death.

Considering serum tropomyosin-specific IgE variation, 100 *μ*l venous blood was obtained from the tail of each mouse weekly after the establishment of an allergic model. Serum was isolated via brief centrifugation of the whole blood. Serum levels of tropomyosin-specific IgE were measured twice by using an ELISA kit. The ELISA plate was washed by using PBS buffer, 200 *μ*l blocking solution was added and incubated for 2 h at 37°C. Horseradish peroxidase- (HRP-) conjugated rabbit anti-mouse secondary antibodies were added. After one-hour incubation at 37°C, TMB (3, 3′, 5, 5′-tetramethylbenzidine) was developed. The absorbance of IgE was measured at 490 nm.

### 2.10. Quantitative PCR Assay

A reverse transcription kit and SYBR Mix was purchased from Takara Company (Dalian, China). Total RNA was isolated by using the RNA isolation kit (Qiagen, Valencia, CA, USA). RNA purity was determined by using NanoDrop2000 (Thermo Scientific Company, Fremont, CA, USA). cDNA was synthesized from one-*μ*g RNA by using a cDNA synthesis kit (Pharmacia Biotech, Piscataway, NJ, USA). Relative mRNA levels of IL-17A, IL-23, Treg-related transcription factor FoxP3, and cytokine TGF-*β* were measured by using quantitative PCR assay via the primers from Table 1. PCR reaction mixtures were set in 20-*μ*l volumes: SYBR Mix 10 *μ*L, primer (*μ*m) 0.4 *μ*L, cDNA 2 *μ*L, and ddH_2_O 7.2 *μ*L. PCR amplification conditions were: 95°C 3 min; 95°C 20 s, 60°C 20 s, 72°C 20 s, and 40 cycles. The relative level of gene expression was calculated by using the 2-AACt method [49].

### 2.11. Measurement of T-Cell Subsets

Rabbit anti-human IgE antibodies were purchased from Southern Biotech (Birmingham, AL, USA); FITC-labeled CD4 (Cat#11-0047-42 for anti-human antibody and Cat# 11-0042-86 for anti-mouse antibody), FoxP3 PerCP-CY5.5 (Cat # 45-4776-42 for anti-human antibody and Cat # 45-5773-82 for anti-mouse antibody), PE-IL-17A (Cat # 12-7178-42 for anti-human antibody and Cat # 25-7177-82 for anti-mouse antibody), and PE CD25 (Cat # 12-0259-42 for anti-human antibody and Cat # 12-0251-82 for anti-mouse antibody) were purchased from American eBioscience Company (San Diego, CA, USA); mouse spleen was sterilely ground to prepare cell suspension. After the cell lysis, the cell concentration was adjusted to 10^7^ cells/ml and incubated with FITC-CD4, PE- CD25, FoxP3 PerCP-CY5.5, and PE-IL-17A. After washing with PBS buffer, the spleen cells were suspended in PBS buffer, and CD4^+^ T-cell subsets of spleen cells were detected by flow cytometry (Beckman Coulter, USA).

### 2.12. Statistical Analysis

All data were presented as mean values ±SD. The chi-squared (*χ*^2^) test was used to compare the number difference between two groups. Student's *t*-test was used to compare mean quantitative differences between two groups. All analyses were performed by using SPSS 20.0 statistical package (IBM Software, NY, USA). The statistical difference was significant if *P* < 0.05.

## 3. Results

### 3.1. Measurement of Food Allergy

Table 2 shows the allergens in the food and the incidence of food allergy varied from 4.3% to 79.3% among 9 kinds of food. All different-age groups were compared with each other, and the statistical difference was insignificant between the group of 4∼7 years old and the group of 8∼12 years old (*P* < 0.05, Table 3).

### 3.2. Clinical Characters

Table 4 shows that there was no significant difference between two groups in age and sex (*P* > 0.05). There was no significant difference in symptoms, allergy grades, and serum IgE (*P* > 0.05). The statistical differences for other parameters were insignificant between two groups, either (*P* > 0.05, Table 4). After one-month follow-up, 2 and 4 persons were withdrawn from BG and CG, respectively. After three-month follow-up, further 3 and 4 persons were withdrawn from BG and CG, respectively.

### 3.3. *B. lactis* Treatment Reduced Allergy Symptoms in the Children with Food Allergy

Allergic symptoms were alleviated in BG when compared with CG (*P* < 0.05, Table 5). After three-month therapy, the therapeutic results for allergic symptoms were still stable and statistical difference was significant between BG and CG (*P* < 0.05, Table 5). Rash grades, eczema scores, abdominal cramps/pain scores, and vomiting grades were reduced significantly after long-term *B. lactis* consumption (*P* < 0.05, Table 5). The levels of serum IgE showed similar results and reduced significantly after long-term *B. lactis* consumption (*P* < 0.05, Table 5).

### 3.4. Serum Levels of IgE in the Children with Food Allergy

**Figure 1:**
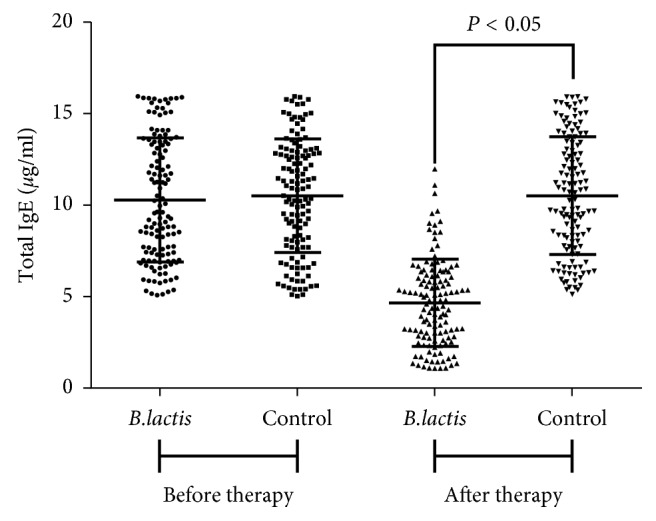
The effects of *B. lactis* on serum levels of IgE in the children with food allergy. *B. lactis* group: the patients received *B. lactis* treatment. Control group: the patients received the solution without *B. lactis* treatment. *n* = 128 for each group. The statistical difference was significant if *P* < 0.05.

Before therapy, the statistical difference for serum levels of IgE was insignificant between control and *B. lactis* groups (*P* > 0.05, Figure 1). After 3-month therapy, the levels of IgE were reduced in the *B. lactis* group and the statistical difference for serum levels of IgE was significant between control and *B. lactis* groups (*P* < 0.05, Figure 1). After 3-month therapy, the statistical difference for serum levels of IgE was significant between control and *B. lactis* groups (*P* < 0.05, Figure 1). All these results indicated that *B. lactis* effectively alleviated the allergic effect by regulating the levels of IgE in the children with food allergy.

### 3.5. *B. lactis* Consumption Increased the Percentage of Treg Cells and Reduced the Percentage of Th17 Cells

**Figure 2:**
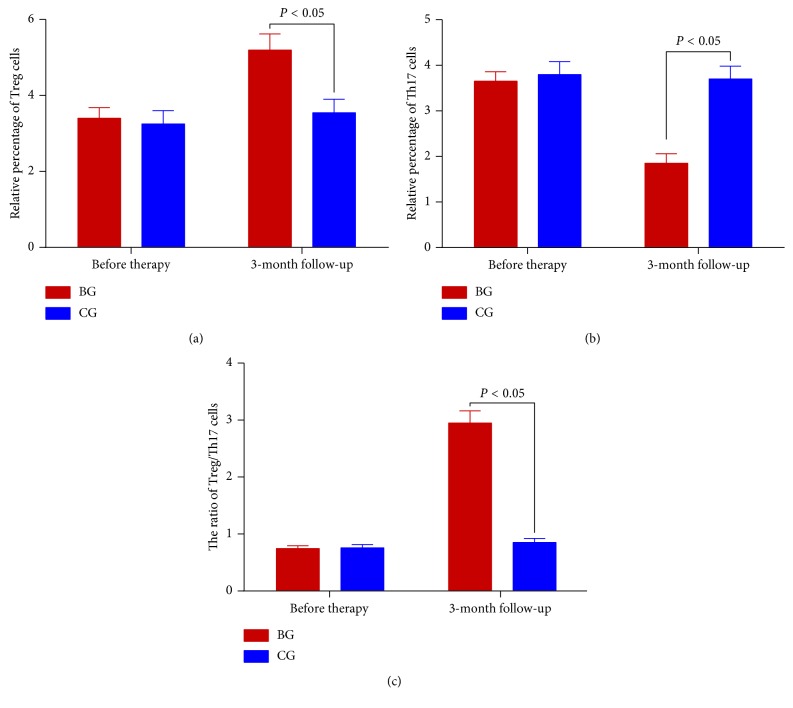
The effects of *B. lactis* on the percentage of Treg cells and Th17 cells in the children with food allergy. (a) The percentage of Treg cells. (b) The percentage of Th17 cells. (c) The ratio of Treg/Th17 cells. The statistical difference was significant if *P* < 0.05.

Before the probiotics consumption, the statistical difference for the percentage of Treg cells (Figure 2(a)) and Th17 cells (Figure 2(b)) and the ratio of Treg/Th17 cells (Figure 2(c)) was insignificant between two groups (*P* > 0.05) in the children with food allergy. After three-month probiotic consumption, *B. lactis* consumption increased the percentage of Treg cells (Figure 2(a)) and reduced the percentage of Th17 cells (Figure 2(b)) and the ratio of Treg/Th17 cells (Figure 2(c)) (*P* < 0.05).

### 3.6. *B. lactis* Consumption Reduced Anaphylaxis Scores in Mouse Allergy Models

**Figure 3:**
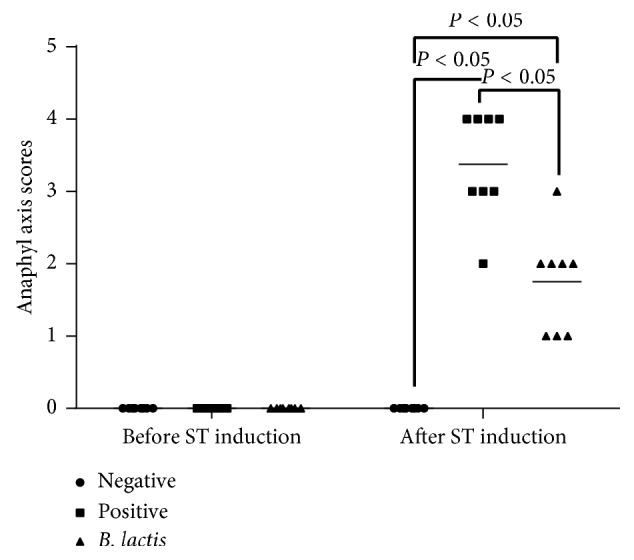
Shrimp tropomyosin-induced anaphylaxis in the mice among different groups. A total of 24 BALB/c mice were randomly divided into 3 groups (*n* = 8 in each group): negative control group (NG), positive control group (PG), and experiment group (EG). The statistical difference was significant if *P* < 0.05.

Allergic symptoms were promoted within 30 min after tropomyosin challenge. The model mice scratched and rubbed around nose, with reduced activity or no activity after prodding. Diarrhea was found after 45-min challenge. After two-hour challenge, anaphylaxis scores were higher in a model group than those in the group treated with *B. lactis* or negative controls (*P* < 0.05, Figure 3).

### 3.7. Serum Levels of IgE in Mouse Allergy Models

**Figure 4:**
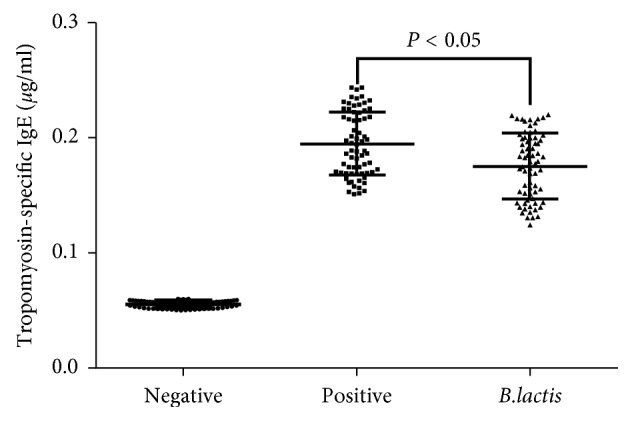
The effects of *B. lactis* on serum levels of IgE in mouse allergy models. BALB/c mice were randomly divided into 3 groups (*n* = 8 in each group): negative control group (NG), positive control group (PG), and experiment group (EG). The mice from PG and EG were individually immunized with tropomyosin and cholera toxin. The mice from NG received cholera toxin in PBS. Subsequently, the mice from EG received *B. lactis*. *n* = 8 for each group. The statistical difference was significant if *P* < 0.05.

To confirm the changes for the serum levels of IgE, an allergy animal model was established. Allergy symptoms were seen from animal models when compared with the negative control group, and both groups have varying degrees of diarrhea. *B. lactis* treatment alleviated allergic symptoms significantly (*P* < 0.05). In the NG, serum levels of IgE (Figure 4) were lowest when compared with other two groups (*P* < 0.05). In the PG, serum levels of IgE (Figure 4) were increased significantly when compared with the NG (*P* < 0.05). After *B. lactis* treatment, serum levels of IgE (Figure 4) were reduced significantly when compared with the PG (*P* < 0.05). *B. lactis* effectively alleviated the allergic responses caused by the original tropomyosin of shrimp in the mouse allergy models.

### 3.8. The Effects of *B. lactis* on Relative mRNA Levels of Treg- and Th17-Related Cytokine

**Figure 5:**
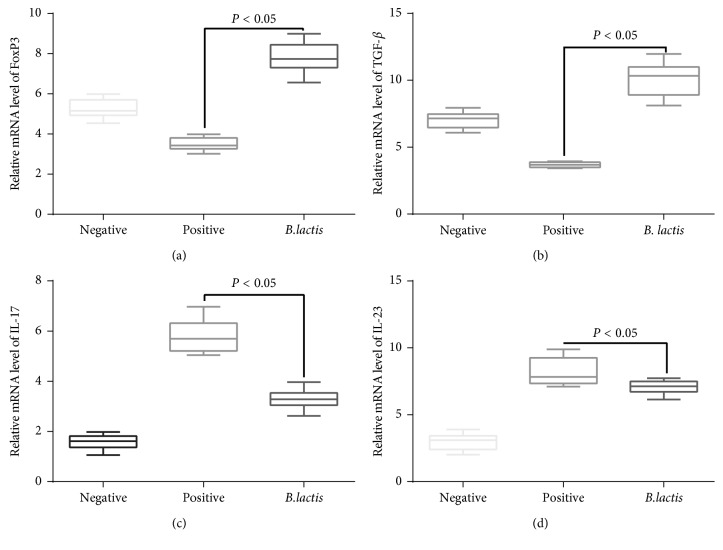
The effects of *B. lactis* on relative mRNA levels of Treg- and Th17-related cytokines in mouse allergy models. (a) The effects of *B. lactis* on mRNA levels of FoxP3 in mouse allergy models. (b) The effects of *B. lactis* on relative mRNA levels of TGF-*β* in mouse allergy models. (c) The effects of *B. lactis* on relative mRNA levels of IL-17A in mouse allergy models. (d) The effects of *B. lactis* on relative mRNA levels of IL-23 in mouse allergy models. The statistical difference was significant if *P* < 0.05.

In the PG, relative mRNA levels of FoxP3 (Figure 5(a)) and TGF-*β* (Figure 5(b)) were lowest when compared with other two groups (*P* < 0.05). In the NG, relative mRNA levels of FoxP3 (Figure 5(a)) and TGF-*β* (Figure 5(b)) were increased significantly (*P* < 0.05). After *B. lactis* treatment, relative mRNA levels of FoxP3 (Figure 5(a)) and TGF-*β* (Figure 5(b)) were increased significantly when compared with the PG (*P* < 0.05). In contrast, relative mRNA levels of IL-17A (Figure 5(c)) and IL-23 (Figure 5(d)) were highest in the PG when compared with other two groups (*P* < 0.05). In the NG, relative mRNA levels of IL-17A (Figure 5(c)) and IL-23 (Figure 5(d)) were decreased significantly (*P* < 0.05). After *B. lactis* treatment, relative mRNA levels of IL-17A (Figure 5(c)) and IL-23 (Figure 5(d)) were significantly reduced when compared with the PG (*P* < 0.05). These results suggest *B. lactis* treatment may affect the balance of Th17/Treg cells since these cytokines are main indicators of the two subsets of T cells. The mouse allergy model may have imbalance of Treg/Th17 cells, and *B. lactis* may regulate the balance. Thus, the effects of *B. lactis* on the balance of Treg/Th17 cells were further explored.

### 3.9. The Effects of *B. lactis* on the Balance of Treg/Th17 Cells

**Figure 6:**
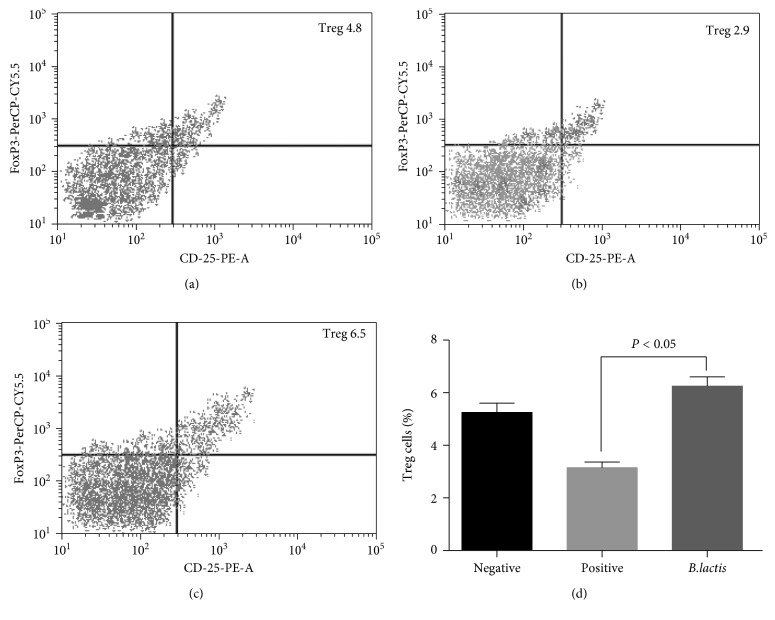
The effects of *B. lactis* on the percentage of Treg cells in mouse allergy models. (a) The percentage of Treg cells in the NG. (b) The percentage of Treg cells in the PG. (c) The percentage of Treg cells in the EG. (d) The effects of *B. lactis* on the percentage of Treg cells. BALB/c mice were randomly divided into 3 groups (*n* = 8 in each group): negative control group (NG), positive control group (PG), and experiment group (EG). The mice from PG and EG were individually immunized with tropomyosin and cholera toxin. The mice from NG received cholera toxin in PBS. Subsequently, the mice from EG received *B. lactis*. CD4^+^ T cells were gated by CD4 expression and forward scatter characteristics. The statistical difference was significant if *P* < 0.05.

**Figure 7:**
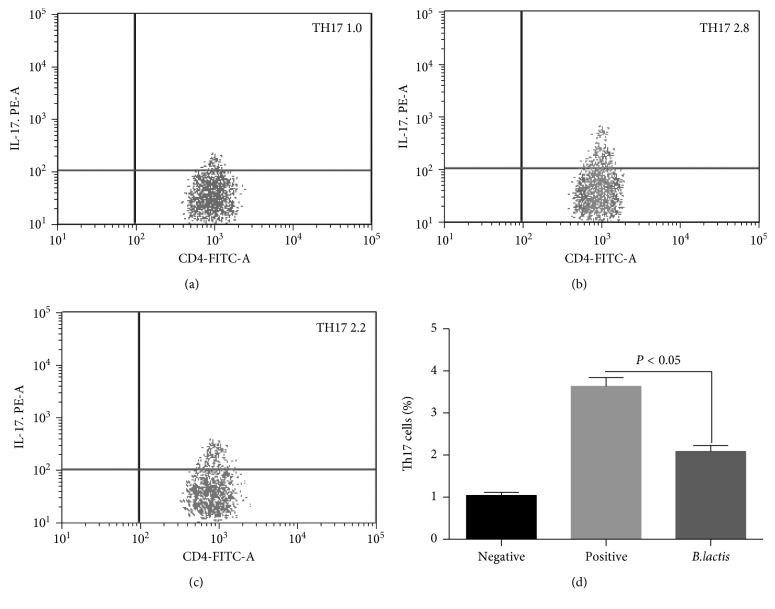
The effects of *B. lactis* on the percentage of Th17 cells in mouse allergy models. (a) The percentage of Th17 cells in the NG. (b) The percentage of Th17 cells in the PG. (c) The percentage of Th17  ells in the EG. (d) The effects of *B. lactis* on the percentage of Th17 cells. BALB/c mice were randomly divided into 3 groups (*n* = 8 in each group): negative control group (NG), positive control group (PG), and experiment group (EG). The mice from PG and EG were individually immunized with tropomyosin and cholera toxin. The mice from NG received cholera toxin in PBS. Subsequently, the mice from EG received *B. lactis*. *n* = 8 for each group. CD4^+^ T cells were gated by CD4 expression and forward scatter characteristics. The statistical difference was significant if *P* < 0.05.

To determine the effect of *B. lactis* on the number of Treg and Th17 cells in children with food allergy, Treg and Th17 cells were measured in three groups.  Figure 6 shows that the percentage of Treg cells in the NG were significantly higher than that in the PG (*P* < 0.05, Figures 4, 4, and 4), indicating that Treg cell differentiation was inhibited in allergy models. After *B. lactis* treatment, the contents were significantly increased when compared with the PG (*P* < 0.05, Figures 4 and 4). The results indicated that *B. lactis* treatment increased the levels of Treg cells.  Figure 7 shows that the percentage of Th17 cells in the NG was significantly lower than that in the PG (*P* < 0.05, Figures 5(a), 5(b), and 5(d)), indicating that Th17 cell differentiation was promoted in allergy models. After *B. lactis* treatment, the percentage of Th17 significantly was reduced when compared with PG (*P* < 0.05, Figures 5(b) and 5(d)). The present findings demonstrated that allergic animal models had less T lymphocytes with Treg cells differentiation and more Th17 differentiation of T lymphocytes. *B. lactis* treatment induced a significant tolerance reaction for allergy symptoms by increasing Treg cells differentiation and suppressing Th17-type cells differentiation.

## 4. Discussion

As people's lifestyle changes [50] and environmental pollution aggravates [51], the therapy of allergic diseases is increasingly challenging and has become a serious disease threatening public health. Food allergies can occur in people of all ages, and high-risk groups are mainly infants and children. Food allergy can cause digestion, skin, nerves, respiratory disorders, and other symptoms [52]. Food allergy is closely related to genetic factors [53], the digestive system [54], environmental elements [55], and gut microbiota [56]. The nine kinds of food in this study are common food in daily life but are susceptible to allergies in children. Egg with high-level protein can cause severe allergy, followed by seafood, beef, milk, and fruit (Table 2). The results are different with previous reports: the allergic events caused by seafood are higher than those caused by eggs [57]. The consumption of eggs, shrimp, and crab is closely related to the environment and life habits, which may be the reason causing the difference.

Different age groups are closely associated with the events of food allergy [58]. The statistical difference for the incidence of allergy symptoms was insignificant between different age groups from 4 to 7 years old and 8 to 12 years old (*P* < 0.05). The age differences for the two groups may not be better criteria for exploring the effects of age on food allergy. Furthermore, nine kinds of common food were selected as targets, but food allergy in children is much more complicated. Further work is highly demanded to address these issues. Food antibodies should be detected as soon as possible if some food allergies were suspected. The children should be prohibited from taking the same food and prevented from receiving further allergic damage and other allergic diseases.

Gut microbiota may also play an important role in egg allergy [59]. Probiotics is a potential way to improve the distribution of gut microbiota [60]. *B. lactis* is a main probiotics in dairy yogurt and has been proved to be effective to improve immune function by improving NK cell function and IFN-gamma levels [61]. The present findings demonstrated that *B. lactis* consumption reduced allergy symptoms of children with food allergy by reducing the serum levels of IgE (Table 5 and Figure 1). There was an improvement in all parameters in BG patients, which was consistent with previous reports. Dietary consumption of *B. lactis* can improve gut microecology and function of the digestive system and reduce the prevalence of rash [62]. Meanwhile, modulation of *B. lactis* for gut microecology may provide an alternative in the prevention of eczema [63]. On the other hand, the consumption of *B. lactis* can reduce abdominal pain (cramps), discomfort, and vomiting symptoms [64, 65]. Furthermore, *Bifidobacterium* species has been reported to inhibit IgE production [66].

Shrimp allergen tropomyosin is one of the main molecules for causing food allergy [10, 67]. According to early reports, shrimp tropomyosin was useful in exploring the mechanisms underlying food allergy in human subjects and assessing efficacy and safety of some therapeutic approaches. Some food allergy models were established by using shrimp tropomyosin [68, 69].

Further experiment showed that *B. lactis* reduced allergy symptoms of animal models with food allergy by reducing the serum levels of IgE (Figure 4). *B. lactis* increased relative mRNA levels of FoxP3 (Figure 5(a)) and TGF-*β* (Figure 5(b)) and reduced the relative mRNA levels of IL-17A (Figure 5(c)) and IL-23 (Figure 5(d)). All these cytokines are associated with the levels of Treg and Th17 cells. Th17 cells are associated with the pathogenesis of Th2-mediated allergic disorders [70]. Th17 cells regulate neutrophil recruitment and play an important role in allergy pathogenesis [71]. Th17 and Treg cells play a critical role in atopic disease [72]. Just as we supposed, *B. lactis* induced a significant tolerance reaction for allergy symptoms by increasing Treg cells differentiation (Figure 6) and suppressing Th17-type cells differentiation (Figure 7). The results were consistent with a previous report that *B. lactis* promoted potentially antiallergenic processes through induction of Th1-type immunity and enhanced the regulatory lymphocyte [39].

There are some limitations for the present work. To maintain the insignificant difference of statistical data between two groups, the both groups were not advised to avoid certain food based on anamnesis and diagnostic test results. Furthermore, the lack of change in scores in the control group did not mean that no effective treatment was offered. The comparison of two groups showed that *B. lactis* treatment would be more effective than control group. The levels of transcription factor FoxP3 and cytokines TGF-*β*, IL-17A, and IL-23 were not measured in the children with food allergy. The work was limited because most patients did not agree to obtain small splenic biopsy by using an invasive surgery. Serum cytokine can be measured simply by using ELISA kits, and the results were unstable with time increasing. The food from different places will result in different allergic symptoms, and the problems were not explored in the present work. The detail components were not analyzed in different kinds of food and can be a main issue affecting the final results. Therefore, further research is highly demanded to address these problems in the future work.

## 5. Conclusion

*B. lactis* can effectively alleviate allergic reactions on food-specific IgE of food in children [73], and its related molecular mechanism may be involved with the balance of the Treg/Th17 cells. *B. lactis* treatment significantly increased the expression of FoxP3 and TGF-*β* related to Treg cells and reduced the expression of IL-17A and IL-23 related to Th17 cells. The probiotics consumption increased the ratio of Treg/Th17 cells to suppress the occurrence of allergic reactions. However, its specific mechanism of action is not yet clear and further study is still needed.

## Figures and Tables

**Table 1:** The primers used for real-time PCR.

Genes	Forward primer sequence (5′-3′)	Reverse primer sequence (5′-3′)
The primers for human		
FoxP3	TTGAACCCCATGCCACCATC	CATCCACCGTTGAGAGCTGG
TGF-*β*	GGGCTTCTCCTACCCCTAC	CTTCCCCTTCTGGGATCTTG
IL-17A	CCCGGACTGTGATGGTCAAC	GGAGGCTCCCTGCGCAGGAC
IL-23	GATGAAGAGACTACAAATG	GAGGCATGAAGCTGGCCCAC
*β*-Actin	GGGCGTGATGGTGGGCATG	TCGGTCAGCAGCACGGGGTG

The primers for mouse		
FoxP3	TTTCCAAGAACGGGCATTA	TGTGGCTGACTGAGGGTGT
TGF-*β*	ACCGCAACAACGCCATCTAT	GCACTGCTTCCCGAATGTCT
IL-17A	AGGGAGAGCTTCATCTGTGG	AGATTCATGGACCCCAACAG
IL-23	TGCTGGATTGCAGAGCAGTAA	GCATGCAGAGATTCCGAGAGA
*β*-Actin	CGCAAAGACCTGTATGCCAAT	GGGCTGTGATCTCCTTCTGC

**Table 2:** Allergic reactions on food-specific IgE of food.

	Egg	Crab	Shrimp	Beef	Cashew	Milk	Mango	Pineapple	Shellfish
Positive number	203	123	134	56	29	21	14	11	79
Percent (%)	79.3	48.0	52.3	21.9	11.3	8.2	5.5	4.3	30.9

**Table 3:** IgE in different age groups.

Parameters	Egg	Crab	Shrimp
4∼7 yr, *n* (%)	127 (62.6)	75 (61.0)	81 (60.4)
8∼12 yr, *n* (%)	76 (37.4)	48 (39.0)	53 (39.6)
Total case, *n*	203	123	134
*χ* ^2^		0.174	
*P* values		0.917	

**Table 4:** Clinical characteristics.

Parameters	BG	CG	*χ* ^2^ and *t* value	*P* value
Gender, male/female	72/56	76/52	0.256	0.613
Age, yr	10.89 ± 3.26	10.23 ± 2.78	−1.313	0.109
Body mass index, weight (kg)/height^2^ (m^2^)	24.67 ± 4.18	24.13 ± 3.95	0.689	0.412

*Allergy symptoms*				
Rash, grades	2.51 ± 1.36	2.62 ± 1.43	−0.718	0.362
Eczema, scores	32.70 ± 11.26	30.54 ± 12.03	−0.964	0.358
Abdominal cramps/pain, scores	22.11 ± 9.34	24.26 ± 8.73	0.648	0.723
Vomiting, grades	1.22 ± 0.87	1.14 ± 0.79	0.452	0.612
IgE (*μ*g/ml)	10.23 ± 5.18	10.36 ± 5.31	0.246	0.482

*Note.* BG, the patients received *B. lactis* treatment; CG, the patients received solution without *B. lactis* treatment. *n*=128 for each group. All data were presented as mean ± SD (standard derivation). The statistical difference was significant if *P* < 0.05 when compared with the control group.

**Table 5:** The effects of *B. lactis* on pediatric allergy.

Parameters	One-month follow-up		*P* value	Three-month follow-up		*P* value
	BG	CG		BG	CG	
*Allergy symptoms*						
Rash, grades	1.51 ± 1.13	2.56 ± 1.67	<0.001	1.46 ± 1.29	2.59 ± 1.85	<0.01
Eczema, scores	11.70 ± 9.36	32.41 ± 14.61	<0.001	11.59 ± 10.88	32.52 ± 15.99	<0.01
Abdominal cramps/pain, scores	8.11 ± 6.73	22.16 ± 8.41	<0.001	6.03 ± 5.26	22.09 ± 11.44	<0.01
Vomiting, grades	1.05 ± 0.97	2.23 ± 1.65	<0.001	1.16 ± 1.08	2.31 ± 1.89	<0.01
IgE (*μ*g/ml)	2.27 ± 1.94	5.82 ± 3.90	<0.001	1.53 ± 1.38	5.96 ± 4.97	<0.01

*Note.* BG, the patients received *B. lactis* treatment; CG, the patients received the solution without *B. lactis* treatment; TSS, total allergy symptom score. *n* = 126 and 124 for BG and CG groups, respectively, after one-month follow-up. *n* = 123 and 120 for BG and CG, respectively, after three-month follow-up. All data were presented as mean ± SD (standard derivation). The statistical difference was significant if *P* < 0.05 when compared with the control group.

## Data Availability

There are no unavailable data for this manuscript. All data are provided in the Results section of this paper.
